# Monthly Summary

**Published:** 1868-02

**Authors:** 


### MONTHLY SUMMARY.

*Ozone.*—The real nature of this curious substance has long been debated. Schœnbein thought it was negative oxygen and called all substances that evolved it *ozonides*. He supposed that there must be an opposite or positive variety of oxygen, which he named *antozone*, and he believed that ordinary oxygen was a compound of these two which mutually neutralized one another. This notion was successfully combatted by Sir B. C. Brodie. In 1860, Andrews and Tait showed that 100 volumes of oxygen subjected to the electric spark, may contract to 92 volumes but never to much less. Hence ozone is denser than oxygen. Furthermore, mercury, introduced into this ozonized oxygen, absorbed ozone, but still left 92 volumes of common oxygen. By heating the ozonized oxygen the 100 volumes were again obtained the ozone being destroyed by heat.

Dr. Odling, starting from the theory that free oxygen consists of two atoms, suggested that these phenomena might be accounted for on the hypothesis that an additional atom of oxygen might be condensed into each molecule, so that the formula for ozone would be $O_3$ and its density one-half greater than that of oxygen. When 100 volumes of oxygen were reduced by ozonization to 92, it might be inferred that 8 volumes of oxygen combined with 16 volumes, and that these 24 volumes were condensed to 16. In this way 8 volumes would seem to disappear. The absorption by mercury might only be the removal of the third atom, or the 8 volumes, so that 92 volumes of common oxygen would remain.

These views have received recently confirmation from the experimental researches of a French chemist, M. Soret. This gentlemen has succeeded in finding, in oil of turpentine, a substance which takes up the whole of the ozone, instead of removing only its third atom. Thus when 92 volumes of ozonized oxygen are mixed with oil of turpentine, a dark white cloud appears; the ozone vanishes, and 76 volumes of common oxygen remains.

---

*Restoration of Patients Under the Influence of Chloroform.*—In a paper read before the British Association, Dr. Richardson stated that the best method to restore a patient about to die from chloroform, was to introduce into the lungs by means of artificial respiration, air heated to 130° F. A bellows connected with a thin coiled tube of platinum, which could be raised to the necessary temperature by a spirit lamp, is the apparatus suggested. The air need only be forced through one nostril.

---

*Cafeine an Antidote to Opium.*—Dr. Henry F. Campbell of Augusta, Georgia, has been experimenting upon cafeine as an antidote to opium, or rather as means of relieving the coma consequent upon the poisonous action of the drug. Dr. C. has published one case of recovery; he now gives us another case, in which, though he failed to restore the patient, he nevertheless produced such effects as to strengthen his confidence in the value of the antidote.

A Jew had swallowed nearly three ounces of laudanum and had been under its influence for fifteen hours. When seen he was blue, with feeble pulse, 100 to the minute, and breathing at the rate of four to the minute. The muscular system was completely relaxed. The treatment consisted mainly of ice to the head and the injection of twenty-five grains of cafeine into the rectum. Under this treatment, the skin resumed its natural tint, tone returned to the muscles, the respiration rose to twenty in the minute, and the man appeared to die from an accumulation of mucus in the bronchi and larynx, the result of previous engorgement. Such effects should certainly lead to further trial of this antidote.

---

*Malformation of the Heart.*—A curious case of malformation of the heart is recorded in the *Medical Times and Gazette* by Mr. Iliffe of Birmingham. A child of four months had been suffering from great dyspnœa accompanied by extreme lividity of the surface. Open foramen ovale was diagnosticated. Upon post-mortem examination, the diagnosis was verified and there was also a small oval aperture of communication between the ventricles. The most remarkable point however was the entire absence of the tricuspid orifice, so that the right ventricle could only get its blood through the abnormal opening between the two ventricles.

---

*New Microscopic Reagent.*—Dr. Schultze, of Rostock, recommends a solution of one part of bichloride of palladium in 800 of water, feebly acidulated with hydrochlore acid, which hardens tissue and facilitates the making of sections. By its use connective tissue remains uncoloured, hyaline membranes assume a light yellow, cell-formations a darker yellow and nerve marrow a greyish black. The fact that unstriped muscular fibre is colored yellow by this reagent, is important, as thereby it is readily distinguished from connective tissue.

*Poison of Syphilis and Gonorrhœa.*—Prof. Salisbury, to whose researches on malarious disease we have already alluded, published in the *American Journal of Medical Sciences* for January, a paper on the poison of these two diseases, in which he maintains that they are caused by the presence of plants of low organization. In the case of syphilis, this algoid vegetation invades the tissues, and finds its way into the blood, while the gonorrhœal plant confines itself to the epithelium. Hence, the writer thinks, the constitutional character of the former disease is accounted for. Figures of these plants, which are extremely minute, are given, but the author strangely neglects to say anything about their size, or to give the microscopic powers used in the observation. He calls the new plants *Crypta syphilitica* and *C. gonorrhœa* (probably *gonorrhœae* or *gonorrhealis* is intended.)

---

*Animal Electricity.*—M. Schultz Shultsenstein has lately published his investigation of the relation of electricity to muscular action. His novel and startling conclusions have been thus formulated:

1. The supposition that living muscle produces electricity is incorrect. If needles be plunged into the foot of a living animal and be placed in connection with a galvanometer, no deflection of the galvanometer needle occurs.

2. Muscles removed from the body give evidence of electricity, but this is because of the combination of the decomposing tissue with the oxygen of the air.

3. Salt water causes the galvanometer needle to be deflected. This explains why salted meat gives evidence of electricity.

4. The supposed electric current in the human muscle is solely caused by the salt water in contact with the tissue.

5. In diseased structures the electric current is derived from the decomposing tissues.

6. The electricity of the secretions is also derived from the decomposing tissues.

7. Animal electricity is an illusion.

The author has requested the French Academy of Sciences to appoint a commission to witness and report on the experiments upon which his conclusions are based.

*Mutability of Species.*—In a recent communication to the Geological Society of Paris, M. A. Gaudry pointed to some striking facts favorable to the theory of the mutability of the species. The sand pits in the environs of Paris, and indeed all drift deposits in general, are very rich in remains of the mammoth or primitive elephant, and of the *elephas antiquus.* These remains chiefly consist of molar or back teeth, in which characteristic differences may be easily recognized. They consequently pertain to two different species, and in order to ascertain whether there exists any close parentage between them, M. Gaudry goes back to the pleistocene period, which lies between the upper tertiary or pliocene, and the drift strata. Now the pleistocene forest-bed of Norfolk, contains a quantity of molars of each of the above species, but it also comprises others slightly differing from both, and also intermediate between those of *elephas antiquus* and *elephas meridionalis*, the latter ceasing to exist when the former and the mammoth begin. These again disappear after the drift, and are followed by other species. Here then we perceive a succession of species, each of which have sprung from the preceding one. During the tertiary period there existed a breed of horses to which palcontologists have given the name of *hipparion;* they had small lateral fingers, thus forming a link between pachydermata and solipedes, which latter was considered perfectly distinct so long as the genus *equus* was characterized by a single finger at each foot. Now, Mr. Owen, on examining the horses' teeth found in the cavern of Oreston, discovered that the *equus plicidens* to which they belonged was intermediate between the hipyarion and the present horse. In the *equus plicidens* the enamel of the teeth presents more folds than in the living breed but in the molars found in our gravel pits, M. Gaudry has perceived gradations between those presenting many and those presenting fewer folds, whence he concludes that our horse is a descendant of the *equus plicidens.* A hippopotamus, the remains of which were discovered at Grenelle a few years ago, appears not to differ materially from the race that now inhabits the rivers of Africa ; and yet at the time the owner of these venerable relics was disporting himself in the Seine, the climate was much colder here than it is now ; so that Mr. Gaudry concludes with great plausibility that, if we had the whole skeleton, some differences would probably appear.

*Compression as a Treatment for Inflammation.*—Beyond its long-established use as a method of treating aneurism, the compression of arterial trunks has lately been considerably employed as a therapeutical resource in the treatment of arthritic inflammations and similar diseases. Vanzetti of Padua (who, we think was the original proposer of this plan of treatment, since adopted in various countries) lately related to the Paris Academy of Medicine some cases of inflammation in which this procedure had been used. The first case was a wound of the hand, attended with excessive inflammation and swelling of the arm, its size being doubled. After twenty-four hours' digital compression of the brachial artery, the limb had returned to its normal size and all the symptoms had been relieved. The cure was complete in two days. The second case was one of malignant pustule upon the fore-arm. The cure of the patient was complete in a month. The third case was of elephantiasis of the leg. Compression of the femoral was kept up at intervals for about two months, when the patient left the hospital considerably improved. Three years after, the patient was seen, and the hypertrophy had entirely disappeared.—*Med. Gazette.*

---

*Wounds Produced by the Chassepot Projectiles*—Dr. Sarazin, of Strasburg, has made some experiments on a corpse at close quarters, with the following results: 1st. The diameter of the orifice made by the bullet entering the body, is not sensibly larger than that of the projectile. 2d. The diameter of the orifice by which the bullet leaves the body, is from seven to thirteen times larger than that of the projectile. 3d. The arteries and veins are cut transversely, retracted and gaping; the muscles torn and reduced to a pulp. 4th. The bones are smashed in a manner out of all proportion with the dimensions of the projectile. To sum up, the wounding effects present a remarkable intensity, and it is well to note that the balls, after passing through the body, pierced two one-inch planks and buried themselves deeply in the wall behind.—*Med. and Surg. Reporter.*

---

*Absorption of Gases by Solids.*—Among the interesting observations of Mr. Graham, Master of the British Mint, upon the passage of liquids and gasses through solids, is the fact that atmospheric air, by passing through india-rubber, becomes super-oxygenated, and will rekindle smoldering wood like pure oxygen. Any kind of light india-rubber receiver, in which a vacum may be obtained, the size being sustained by mechanical means, will collect super-oxygenated air ; the better if the india-rubber be thin and the temperature high. Mr. Graham makes the suggestion that the solid films pass gases through them by first condensing them to a liquid form within the substance, and then passing them off on the other side by evaporation. Hydrogen passes through red-hot platinum, while oxygen and nitrogen do not, or not in appreciable quantities ; hence their compounds with hydrogen are readly dialysed by this method. The passage of carbonic acid, chlorine, hydro-chloric acid, vapor of water, ammonia, coal gas, and hydro-sulphuric acid, is also inappreciable, while the hydrogen, in compounds containing it, passes. One volume of red hot platinum absorbed 0.207 volume of hydrogen, retained it while cold, and gave it off on reheating. One volume of palladium absorbed 643 volumes of hydrogen, sensibly increasing its weight, and when heated afterward, gave off the most or it in a continuous stream. On the other hand, osmium-iridium does not absorb hydrogen, and copper absorbs it very slightly. Gold absorbs hydrogen and nitrogen slightly. Silver absorbs 0.289 of its volume of hydrogen, and then presents a beautifully frosted appearance. Oxygen is taken up in the proportion of 0.745. Red-hot iron and steel pass hydrogen as readily as platinum does.

---

*Oxygen in the Market.*—A company has been formed in Paris under the style of Jos. de Susini & Co., for the manufacture and sale of oxygen to be mixed with ordinary illuminating gases. The calculation is that an addition of one-third oxygen will be equivalent to multiplying a given quantity of illuminating gas eight times, the price of oxygen being fixed at only 2½ times that of ordinary gas. The superoxygenated gas will be used in lighting the International Lecture-room of the Exposition.

---

*Disease Produced by Sleeping Together.* During the night there is considerable exhalation from our bodies, and at the same time we absorb a large quantity of the vapors of the surrounding air.

Two healthy young children sleeping together will mutually give and receive healthy exhalations; but an old, weak person near a child will, in exchange for health, only return weakness. A sick mother near her daughter communicates sickly emanations to her; if the mother has a cough of long duration, the daughter will at some time also cough and suffer by it; if the mother has pulmonary consumption, it will be ultimately communicated to her child. It is known that the bed of a consumptive is a powerful and sure source of contagion, as well for men as for women, and the more so for young persons. Parents and friends ought to oppose as much as in their power the sleeping together of old and young persons, of the sick and of the healthy. Another reason ought to forbid every mother or nurse keeping small children with them in bed; notwithstanding the advice of prudence, no year passes that we do not hear of a new involuntary infanticide. A baby full of life, health and vigor in the evening is found dead the next morning, suffocated by its parents or nurse.—*Exchange.*—*St. Louis Med. Reporter.*

---

*Re-vaccination; value of the Scar left by the first Vaccination as an Indication of its Protective Influence.*—We find the following interesting and important statistics in the Report of Drs. Seaton and Buchanan on the State of Public Vaccination in London, and on the recent Epidemic of Smallpox, appended to the Sixth Report of the Medical Officer of the Privy Council for 1863.

" In the course of our school inquiry, we obtained facts that corroborate in the strongest way the law of connection between deficient vacine scars and post-vaccinal smallpox. By showing how much smallpox has prevailed in the vaccinated children, the facts we are going to cite would be of themselves a sufficient condemnation of much of London vaccination. We found 88 children scarred by smallpox out of the 49,570 school children who bore vaccine scars. This is at the rate of 1.78 per 1000 of the vaccinated children. Excluding the infant schools, and looking only to those children whose ages have given them longer exposure to smallpox, it was found (1), with respect to the quality of the vaccine scar, that out of each thousand children with typical scars, 1.22 were pitted by smallpox; out of a thousand with tolerable scars, 2.35 were pitted by smallpox; and out of a thousand with bad marks, 7.60 were pitted by smallpox. (2.) As for the protective influence of the quantity of vaccination in the individual, it was further ascertained that of those children who had 4 scars (whatever their quality,) 0.67 only per thousond were pitted by smallpox; of those who had 3 scars, 1.42 in the thousand were pitted; of children with 2 marks, 2.49 in the thousand were scarred by smallpox: while those children who had only one vaccination mark were scarred with smallpox at the rate of 6.80 in the thousand. At the one extreme of goodness, with 4 more typical scars, only 0.62 per thousand children were scarred by smallpox, while at the other extreme of badness, with one bad scar only, 19.0 per thousand were scarred by smallpox. The best vaccination, therefore, was more than 30 times as protective as the worst."—*Boston Medical and Surgical Journal.*

---

*Weight of the Human Brain*—In the *Archiv fur Anthropologie*, Dr. A. Weisback has been giving several articles on the relative weight of the brains of the populations of the Austrian empire, in respect to the bodily size, age, sex, and disease. Some of his conclusions are as follows:

"Among Germans 20 years of age, those of medium height have the largest brains.

"With increasing size the cerebellum increases, while the cerebrum relatively decreases.

"In chronic sickness the total weight of the brain decreases but the decrease is confined to the cerebrum and the pons, the cerebellum relatively increasing.

"The total weight of the brain, and the actual weight of the cerebrum are greatest at about the age of thirty, from which period both steadily decrease until at the age of 80, ten per cent. is lost.

"The pons varolii increases to the fiftieth year, and then steadily decreases, sometimes 17 per cent. in a decade.

"On the whole the female brain is smaller than the male, but in certain races this difference is confined to the posterior, in others to the anterior segment.—*Med. and Surg. Reporter.*

